# Comparative Genomic Analyses of *Flavobacterium psychrophilum* Isolates Reveals New Putative Genetic Determinants of Virulence Traits

**DOI:** 10.3390/microorganisms9081658

**Published:** 2021-08-03

**Authors:** Daniel Castillo, Valentina L. Donati, Jóhanna Jørgensen, Krister Sundell, Inger Dalsgaard, Lone Madsen, Tom Wiklund, Mathias Middelboe

**Affiliations:** 1 Marine Biological Section, Department of Biology, University of Copenhagen, 3000 Helsingør, Denmark; daniel.castillo@bio.ku.dk (D.C.); johannajorgensen88@gmail.com (J.J.); 2 Instituto de Investigación Interdisciplinar en Ciencias Biomédicas SEK (I3CBSEK), Universidad SEK, Santiago 7520317, Chile; 3 Unit for Fish and Shellfish Diseases, National Institute of Aquatic Resources, Technical University of Denmark, 2800 Kongens Lyngby, Denmark; valdo@aqua.dtu.dk (V.L.D.); inda@aqua.dtu.dk (I.D.); loma@aqua.dtu.dk (L.M.); 4 Laboratory of Aquatic Pathobiology, Environmental and Marine Biology, Åbo Akademi University, 20500 Turku, Finland; Krister.Sundell@abo.fi (K.S.); tom.wiklund@abo.fi (T.W.)

**Keywords:** *Flavobacterium*, pathogenicity, pan-genome, virulence, evolution, pathogen, genomics, freshwater, T9SS

## Abstract

The fish pathogen *Flavobacterium psychrophilum* is currently one of the main pathogenic bacteria hampering the productivity of salmonid farming worldwide. Although putative virulence determinants have been identified, the genetic basis for variation in virulence of *F. psychrophilum* is not fully understood. In this study, we analyzed whole-genome sequences of a collection of 25 *F. psychrophilum* isolates from Baltic Sea countries and compared genomic information with a previous determination of their virulence in juvenile rainbow trout. The results revealed a conserved population of *F. psychrophilum* that were consistently present across the Baltic Sea countries, with no clear association between genomic repertoire, phylogenomic, or gene distribution and virulence traits. However, analysis of the entire genome of four *F. psychrophilum* isolates by hybrid assembly provided an unprecedented resolution for discriminating even highly related isolates. The results showed that isolates with different virulence phenotypes harbored genetic variances on a number of consecutive leucine-rich repeat (LRR) proteins, repetitive motifs in gliding motility-associated protein, and the insertion of transposable elements into intergenic and genic regions. Thus, these findings provide novel insights into the genetic variation of these elements and their putative role in the modulation of *F. psychrophilum* virulence.

## 1. Introduction

*Flavobacterium psychrophilum* is a yellow-pigmented, Gram-negative fish pathogenic bacterium with a global distribution in freshwater aquaculture, and causing “bacterial cold water disease” (BCWD) and “rainbow trout fry syndrome” (RTFS) [1]. The disease results in high rates of fry mortality, increased tendency for other infections, and high costs of treatment with antibiotics; causing important economic losses for salmonid aquaculture worldwide [2]. A diverse array of visible phenotypic alterations are attributable to *F. psychrophilum* infection, including necrotic lesions, partial dark skin coloration, exophthalmia, anemia, ascites, and vertebral deformities of the fish [1,2]. These signs have been related to the presence of extracellular proteases [3], adhesion or biofilm formation [4], hemolysis [5], and secreted systems [6,7]. Historically, the initial isolation of *F. psychrophilum* was reported in the USA in the 1940s [1]; since then, *F. psychrophilum* isolates have been obtained from all salmonid producing countries, including European nations [8,9,10].

Previous studies on *F. psychrophilum* genomics have mainly focused on describing the genomic properties involved in genetic diversification and virulence. First, whole genomic analyses displayed a well conserved core genome with high similarities at nucleotide level in isolates obtained across large geographical scales, suggesting that specific genomic differences in *F. psychrophilum* isolates are mainly driven by gain and loss of mobile genetic elements (MGE) [8,11]. Second, pathogenic-related genes associated with functions such as proteases, adhesion, transport, and motility were found in a collection of isolates from Denmark, Chile, and the USA [11]. Third, genome analyses suggested that *F. psychrophilum* uses the widespread type IX secretion system (T9SS) to secrete many surface adhesins, soluble or cell-associated peptidases, nucleases, and other hydrolytic enzymes [6,7,12,13]. Finally, the genomes of 41 *F. psychrophilum* isolates displayed the presence of small plasmids and genomic islands encoding DNA replication, restriction-modification systems, phage-related elements, and transcriptional regulator genes [8]. Together, these findings suggested that the genomic contents could provide valuable insights into the mechanisms of pathogenicity in *F. psychrophilum*. However, despite the global picture of genomic diversity obtained for this pathogen (>160 genomes available in public databases in 2021), there are still gaps in our understanding of the links between specific genotype and virulence characteristics of *F. psychrophilum*, which are required to unravel the molecular evolution of this pathogenic bacterium. Thus, combining comparative genomics with virulence assays could provide an excellent approach to obtain a more detailed characterization of the gene repertoire or genetic regions that undergo variation (e.g., clusters of tandem repeats genes and amino acid sequences) in relation to changes in virulence properties. Linking potential genomic and phenotypic patterns could contribute to disease prevention approaches, including alternative methods such as phage therapy [14,15,16,17].

Studies on genetic variants of fish pathogenic bacteria have demonstrated that the evolution of virulence traits includes several factors such as acquisition of genetic elements by horizontal gene transfer [18], genetic microvariations [19], and genome reduction and gene loss [20]. In this study, we compared the genomic sequences of twenty-five *F. psychrophilum* isolates collected in rainbow trout farms around the Baltic Sea (Denmark, Finland, Sweden, Germany, Russia, and Poland), previously characterized phenotypically by Sundell et al., 2019 [4]. Thus, we explored the relationship between genomic diversity and virulence traits among the *F. psychrophilum* isolates. Together, these approaches provided insights into the local genetic and pathogenic evolution of the fish pathogen *F. psychrophilum* in the Baltic Sea rainbow trout production.

## 2. Materials and Methods

### 2.1. Strain Isolation, Medium Composition, and Growth Conditions

This study used twenty-five *F*. *psychrophilum* isolates from rainbow trout fish farms in different geographic localities in Denmark, Finland, Sweden, Germany, Poland, and Russia, covering a spatial scale of >2000 km and a temporal scale of >22 years (Table S1) [4]. For comparative purposes, the type strain NCIMB 1947^T^ was also included in this study [21]. The isolates were stored at −80 °C in TYES broth (tryptone 0.4%, yeast extract 0.04%, CaCl_2_ · 2H_2_O 0.05%, and MgSO_4_ · 7H_2_O 0.05%) with 30% glycerol [22]. For culturing the *F. psychrophilum* isolates, cells from the −80 °C stocks were inoculated in 50 mL TYES broth and incubated at 15 °C with agitation (200 rpm) for 48–72 h [11].

### 2.2. DNA Extraction

DNA from *F. psychrophilum* isolates were extracted from cells harvested by centrifugation (5000× *g*, 15 min, 4 °C) using a Wizard^®^ Genomic DNA Purification Kit (Promega; Catalogue number A1120). The bacterial DNA of the four isolates selected from the Pacific Biosciences (PacBio) sequencing platform was extracted from 48 h-old bacterial broth cultures using a QIAamp DNA Mini Kit (Qiagen; Catalogue number 51306). The amount of genomic DNA was measured using a Quant-iTTM PicoGreen^®^ quantification kit (Invitrogen, Waltham, MA, USA).

### 2.3. Genome Sequencing, Assembly, and Annotation

The genomic DNA sequences of twenty-five *F*. *psychrophilum* isolates were obtained using the Illumina HiSeq platform at the FIMM Technology Centre (Finland) and BGI (China) with pair-end read sizes of 100 bp. Library construction, sequencing, and data pipelining were performed in accordance with the manufacturer’s protocols. The Illumina data were assembled into contiguous sequences using Geneious software version 10 [23], then, short and low coverage contigs were filtered out. The remaining contigs were aligned using the previously sequenced *F. psychrophilum* strain JIP02/86 as a reference genome (GenBank accession number: AM398681; September 2019) [3]. In addition, plasmid sequences were identified (3.3 kb and 2.1 kb respectively; Table S1) that did not align with the reference genome. In addition, *F. psychrophilum* isolates FPS-R7, FPS-S6, 950106-1/1, and 160401-1/5N were selected for sequencing using the Pacific Biosciences (PacBio) sequencing platform (BGI, China). Library construction, sequencing, and data pipelining were performed in accordance with the manufacturer’s protocols. A hybrid assembly was made using Ilumina (100 bp) and PacBio read data (average 20 kb) by the Flye assembler program [24]. Circularized assemblies were further polished with the BUSCO [25] and CheckM [26] packages to correct possible single-base and indel errors. To trace the presence of any plasmid, the filtered reads were mapped using SOAP to the bacterial plasmid database [27]. Annotation of the genomic sequences was done using the NCBI Prokaryotic Genome Automatic Annotation Pipeline (PGAAP) [28].

### 2.4. Predictions of Genomic Islands, Virulence-Related Factors and Prophages

We used islandviewer v.4 [29] and MAUVE [30] to predict the putative genomic islands (GIs) (>8 kb, >8 ORFs, associated with integrases or transposases). A virulence database was constructed for *F. psychrophilum*, containing all the putative virulence-related factors recognized previously [3,8,11] and new virulence genes identified by searching against MvirDB (E-value ≥ 10^−5^; identity ≥ 35%; coverage ≥ 75%) [31], virulencefinder 1.2 [32], and RAST [33]. Prophage-related sequences were identified by running bacterial genomes in PHASTER [34]. Putative subcellular localization of ORFs was performed using server tools. Prediction of the localization of bacterial proteins was achieved using PSort V3.0b.75 [35]. Checking of trans-membrane helices (TMH) was performed with TMHMM V2.0c.76 [36]. Predictions of signal peptides were obtained using SignalIP V3.0.77 [37]. Detection and alignment of repeats in protein sequences was accomplished using motif scan [38].

### 2.5. Pan Genome Analysis

In order to predict the genomic diversity in *F*. *psychrophilum*, the bioinformatics program EDGAR [39] was used to predict the pan genome of all twenty-five *F*. *psychrophilum* isolates and calculate the pan-genome (total gene repertoire), dispensable genome (genes found in two or more genomes but not in all the sequences), accessory genome (specific genes, only found in one genome), and core genome (common genes, mutually conserved). Pan-genome development was calculated by iterative pairwise comparison of *F. psychrophilum* genomic sequences. Using the metacontig function of EDGAR, we also defined custom groups of *F*. *psychrophilum* genomes for which the core genome or the pan genome were stored as virtual contigs [11].

### 2.6. Phylogenetic Analysis

To determine the phylogenetic relationship among *F*. *psychrophilum* isolates based on genomic data, we selected a set of orthologous genes shared by all twenty-five isolates (1866 genes present in a single copy, paralogs not included) using OrthoMCL with an e-value cut off 10^−8^ [40]. The set of 1866 single core and virulent-related genes were first aligned at amino acid level using Clustal W version 2.0 [41]. The alignment of all amino acid sequences from orthologous genes was concatenated using FASconCAT [42]. A gene tree was constructed using PhyML [43].

### 2.7. Accession Numbers

Accession numbers for the twenty-five *F. psychrophilum* isolates and their respective plasmid sequences are listed in Table S1.

## 3. Results

### 3.1. Virulence Properties of F. psychrophilum Isolates

We obtained twenty-five *F. psychrophilum* isolates from infected rainbow trout [4]. The *F. psychrophilum* isolates were obtained from spleens and kidneys (Table S1). The type strain NCIMB 1947^T^ was also included in this study as a reference strain. In vitro proteolytic activities (collagenase, elastinase, caseinase) and gliding motility were previously measured for all the isolates [4]. Moreover, the virulence phenotype by median lethal dose (LD_50_) of this entire *F. psychrophilum* strain collection had been previously reported [4], and represents a more direct infection process driven by *F. psychrophilum*. The challenge trials (intramuscular injection) with juvenile rainbow trout (mean weight 5 g) divided the isolates into four groups referred to as high (7 isolates; LD_50_ < 10^5^), moderate (13 isolates; LD_50_ = 10^5^–10^6^), weak (5 isolates; LD_50_ > 10^6^), and non-virulent (1 isolate; no mortality observed) (Table S1) [4].

### 3.2. Genomic Characteristics of F. psychrophilum Isolates

The genomic annotations obtained from the twenty-five *F. psychrophilum* isolates were analyzed together with the genomic annotation of the type isolate NCIMB 1947^T^ [17]. The isolates varied in genomic size from 2.71 to 3.20 Mb, with a GC content from 32.3% to 32.6% (Table 1). Analysis of annotated sequences revealed relatively similar coding sequences (CDS) among all the isolates, ranging from 2258 to 2806 (Table 1). One plasmid of either 3.3 or 2.1 kb was present in 15 out of 26 *F. psychrophilum* isolates (Table 1). In contrast to a previous genomic analysis of the type strain NCIMB 1947^T^ [8], we did not identify plasmids in this strain in this study, suggesting that the plasmids may have been unstable and lost from the cell.

### 3.3. Relation of F. psychrophilum Pan Genome and Virulence Traits

**Figure 1:**
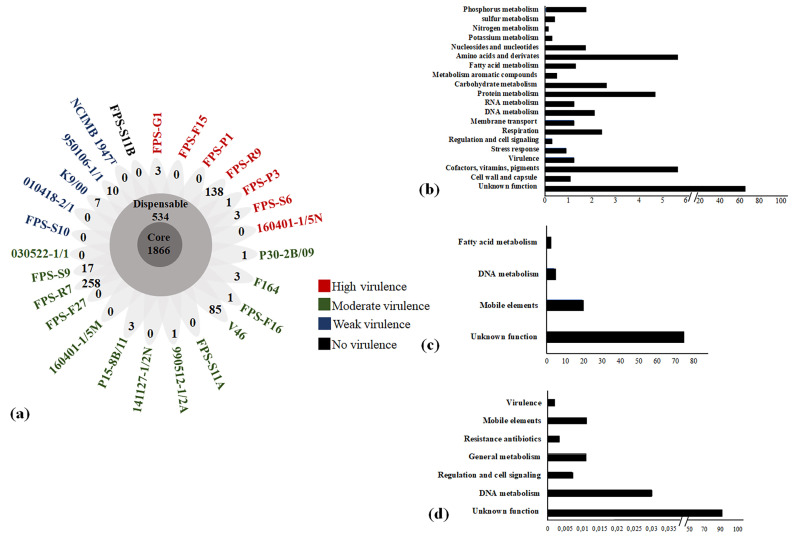
Pan-genome of *F. psychrophilum* isolates. (**a**) The flower plot represents the number of shared (core), dispensable, and accessory genes based on cluster orthologs for each DNA sequence. Petals display numbers of strain-specific genes (accessory genome) found in each genome of *F. psychrophilum*. The array of colors indicates the virulence category as found in the juvenile rainbow trout model [4]. (**b**) COG subcategories of predicted genes within the core genomes of *F. psychrophilum* isolates. Each category is graphed as a percentage of the total number of genes in the core pool of genes. (**c**) COG subcategories of predicted genes within the dispensable genome of *F. psychrophilum* isolates. Each category is graphed as a percentage of the total number of genes in the dispensable pool of genes. (**d**) COG subcategories of predicted genes within the accessory genome of *F. psychrophilum* isolates. Each category is graphed as a percentage of the total number of genes in the accessory pool of genes.

In order to examine the gene repertoire of the twenty-six *F. psychrophilum* isolates (including the type strain NCIMB 1947^T^), a pan genomic analysis was made using the EDGAR platform (Figure 1; Figure S1). The pan genome increased slightly with each addition of a new genome and had at least 4550 ORFs (Figure S1). In contrast, the core genome decreased with the addition of each new genome, reaching 1866 ORFs (Figure S1; Figure 1a). These core-related genes were allocated to putative functional categories using the Clusters of Orthologous Groups of Proteins (COG) database (Figure 1b). The results showed that approximately 65% of ORFs were assigned as hypothetical proteins, 5.6% to cofactor and amino acid derivates, 4.7% to protein metabolism, and 1.2% to putative virulence-related proteins (Figure 1b). Moreover, the dispensable genome (shell genes present in two or more isolates) reached 534 ORFs and was split between 20% mobile elements (plasmids and prophages) and 13% metabolic-related proteins (Figure 1c). Finally, the remaining 529 ORFs were defined as the *F. psychrophilum* accessory genome (Figure 1a). The number of nonduplicated unique genes in each *F. psychrophilum* isolate varied from 0 to 258 ORFs. The *F. psychrophilum* isolates FPS-R7, FPS-R9, and V46 had the largest numbers of accessory genes, being 258, 138, and 85, respectively (Figure 1a). Functional annotation showed that 91% of ORFs were classified as hypothetical proteins (Figure 1c).

**Figure 2:**
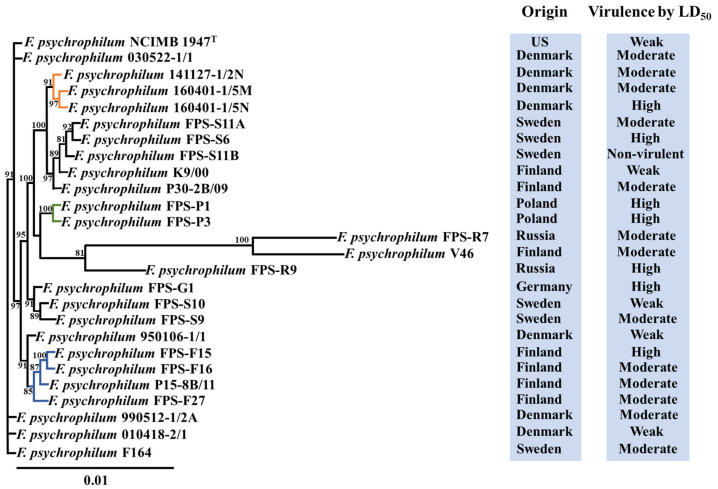
Core genome phylogeny of *F. psychrophilum isolates*. The maximum likelihood tree was obtained from a concatenated amino acid sequence alignment of the orthologous core genes (1866 ORFs) for the twenty-five *F. psychrophilum* isolates and the type strain NCIMB 1947^T^. The numbers above the branches indicate the bootstrap value. Bootstrap values of <80% were removed from the tree. The horizontal bar at the base of the figure represents 0.01 substitution per amino acid site. The virulence properties of the isolates and geographical places of origin were added to facilitate comparison.

Following the analysis of the *F. psychrophilum* pan genome, we examined possible correlations between the virulence traits and genomic repertoire. First, we inferred the phylogeny of our *F. psychrophilum* strain collection by comparing 1866 core ORFs for each genomic sequence (Figure 2). The phylogenetic tree revealed distinct clustering of the isolates, without clear correlations with the virulence properties or country of isolation (Figure 2). For example, *F. psychrophilum* isolates FPS-G1, FPS-S10, and FPS-S9 clustered together, but originated from two different countries and showed high, moderate, and low virulence, respectively (Figure 2; Table S1). However, some isolates clustered according to the geographical origin of isolation such as: Danish isolates 141127-1/2N, 160401-1/5N, and 160401-1/5M; Polish isolates FPS-P1 and FPS-P3; and Finnish isolates FPS-F15, FPS-F16, FPS-F27, and P15-8B/11 (Figure 2; highlighted colors). In addition, we investigated the dynamic nature of virulence-related gene repertoires across the *F. psychrophilum* isolates. One hundred and nine virulence-related genes had been previously identified in *F. psychrophilum* isolates (Table S2). More than 99% of these virulence-related genes were present in all isolates (Table S2). Genes involved in gliding motility, T9SS, metalloproteases, stress response, adhesion, and virulence were found in all isolates (Table S2). For example, we found a broad distribution of genes whose virulence had previously been confirmed in in vivo experiments: exbD2 (id: E5164_11170) of a TonB system [44], collagenase (id: E5164_07005) [45], fpgA type-2 glycosyltranferase (id: E5164_06305) [46], and the thiol oxidoreductase-like tlpB (id: E5164_02660) [47]. Interestingly, the Swedish isolate FPS-S11B revealed a single mutation in the gliding motility gene gldB (id: H0I52_08290), causing a premature stop codon in the amino acid sequence, which was previously linked to a complete loss of virulence [12].

**Figure 3:**
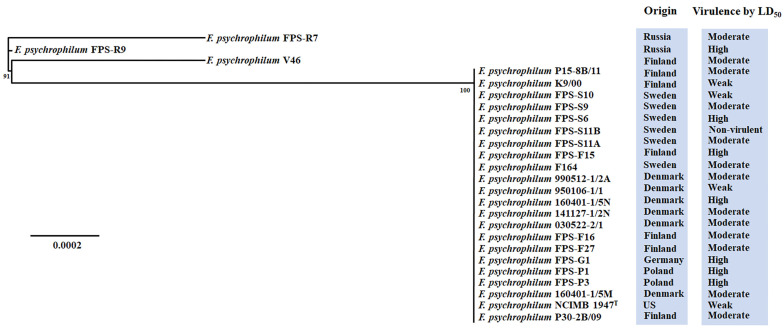
Phylogenetic tree of 109 concatenated virulence-related factors shared by all *F. psychrophilum* isolates. The phylogenetic tree was constructed based on the maximum likelihood algorithm, using a concatenated alignment of 109 amino acid sequences inferred from putative virulence-related factors identified in the pan genome analysis (Table S2). The numbers above the branches indicate the bootstrap value. The horizontal bar at the base of the figure represents 0.0002 substitutions per amino acid site. The virulence ranking and origin of isolation for each isolate was added to facilitate comparison.

On the other hand, only the high-virulent isolates FPS-P1, FPS-R9, and FPS-P3, and the moderate-virulent isolate FPS-R7 had a multicopper oxidase (id: QRE04907.1). Moreover, the moderate-virulent isolates V46 and FPS-R7 harbored a multi antimicrobial extrusion pump (id: QRE04889.1) (Table S2). To reveal the evolution of the virulence, we inferred the genetic diversity of 109 representative virulence-related factors shared by the *F. psychrophilum* isolates (Figure 3). Only the high-virulent isolate FPS-R9 and moderate-virulent isolates V46 and FPS-R7 tended to cluster as mono-phylogenetic groups (Figure 3). The remaining isolates, which presented from weak- to high-virulent phenotypes clustered together in the same phylogenetic group (Figure 3). These findings suggest an unclear association between virulence traits and well-shared virulence-related gene content in *F. psychrophilum*.

Similarly, the dispensable genome did not indicate a coherent correlation with virulence traits. For example, two plasmids (3.3 and 2.1 kb) encoding a toxin-antitoxin system were found in both virulent (e.g., FPS-G1, FPS-F15) and weak or non-virulent isolates (950106-1/1 and FPS-S11B, respectively) (Table 1; Figure S2). Similarly, a 6H-like prophage element was found in seven out of 26 isolates (Table S1) [48]. On other hand, only high-virulent isolates FPS-G1 and FPS-P1 had a genomic island associated with transposase and tRNA-Asn (Figure S2).

Finally, there was no clear link between virulence traits and the number of accessory genes. Instead, the virulence tended to be distributed among the different *F. psychrophilum* clusters (Figure 1a). For example, isolates FPS-G1 and FPS-F15 showed the highest virulence in the juvenile rainbow trout infection model and contained only three and zero accessory genes, respectively (Figure 1a). Contrarily, *F. psychrophilum* isolate FPS-R7, ranked as one of the lowest moderate-virulent isolates, had 258 accessory genes (Figure 1a). Interestingly, this specific isolate had ten genomic islands, being the GI-10 with a length of around 320 kb and encoding multicopper oxidase (id: H0H26_04775), cysteine protease (id: H0H26_04400), β-lactamase (id: H0H26_04585), multidrug efflux pumps (id: H0H26_04095- H0H26_04110), and multiple hypothetical proteins (Table S3).

### 3.4. Precision Long-Read Sequencing for F. psychrophilum

Long-read sequencing approaches can produce completely closed genomes, which allow the assembly of complex genomic areas (e.g., transposon elements and highly repetitive regions) [49] and provide an opportunity to identify new genetic elements involved in virulence mechanisms, besides the gene loss function from single mutations. By combining shot-read Illumina and long-read Pac Bio technologies (hybrid assembly), we fully sequenced four *F. psychrophilum* isolates, which displayed different virulence phenotypes in the juvenile rainbow trout infection model. The selected *F. psychrophilum* isolates were the high-virulent isolates FPS-6 and 160401-1/5N, the moderate-virulent isolate FPS-R7 and the weak-virulent isolate 950106-1/1 (Table S1). The non-virulent isolate FPS-S11B was not included in the strains selected for Pac Bio sequencing as the loss of virulence in this strain had previously been identified to be caused by a single mutation in the gene gldB [12].

**Figure 4:**
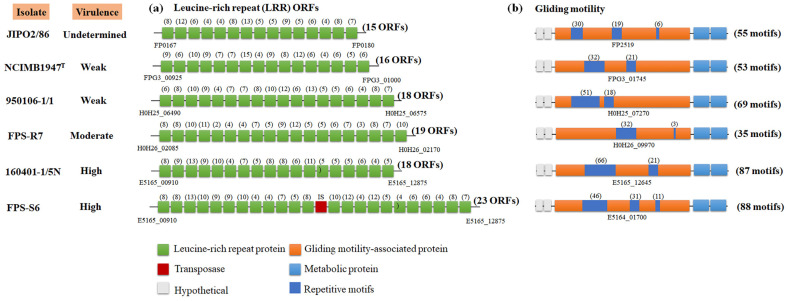
Schematic distribution of repetitive genetic regions in *F. psychrophilum* isolates. (**a**) Localization of leucine-rich repeats identified by hybrid assembly of Illumina and PacBio sequencing technologies. (**b**) Representative amino acid repeat units in gliding motility-associated protein. Genes are represented as squares. Loci tags, isolates, and virulence phenotype are shown in the figure to facilitate comparison. The number of repetitive motifs is presented in parenthesis and their amino acid sequences are described in the Table S4.

For Illumina data, we mapped the reads to the published reference genome *F. psychrophilum* JIP02/86 (Figure S3; see materials and methods). For all the isolates, we identified two genetic zones with very high coverage (from 1500× to 2200×) caused by repetitive DNA sequences, which had around 10× more coverage than the entire genome (Figure S3). The first zone was in a genetic region of 21 kb, which had leucine-rich repeat (LRR) proteins (Figure S3A), and the second region encoded a putative gliding motility-associated protein (Figure S3B). Based on the hybrid analysis, the *F. psychrophilum* isolates FPS-R7, FPS-S6, 950106-1/1, and 160401-1/5N showed a different number of LRRs in comparison to the reference strain JIP02/86 (Figure 4). For example, the high virulent isolates FPS-S6 and 160401-1/5N harbored 23 and 19 LRR proteins, respectively, and these isolates tended to have a higher number of LRRs than the moderate- and weak-virulent isolates FPS-R7 and 950106-1/1, which had 19 and 18 LRR ORFs, respectively, and the weak-virulent strain NCIMB 1947^T^, which had only 16 LRR ORFs (Figure 4a). Moreover, the putative gliding motility protein varied in repetitive motifs among the *F. psychrophilum* isolates (Figure 4b; Table S4). The high-virulent isolates FPS-S6 and 1060401-1/5N had higher numbers of repetitive units, with a total of 87 and 88, respectively, whereas the moderate isolate FPS-R7 and the weak-virulent isolate 950106-1/1 had 35 and 69 units, respectively. The weak-virulent strain NCIMB 1947^T^ had 55 motifs (Figure 4b).

**Figure 5:**
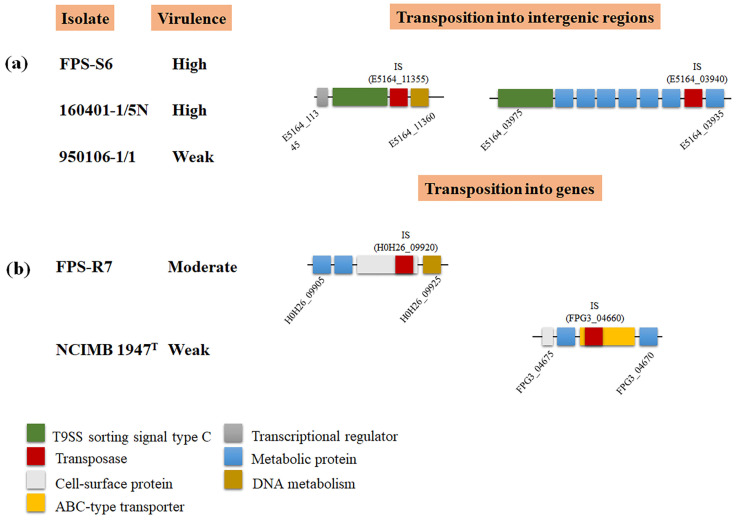
Selected examples of transposon insertion in *F. psychrophilum* isolates. (**a**) Example of IS transposition into intergenic regions. (**b**) Example of IS in encoding genetic regions. Genes are represented as squares; insertions of transposases into genes are represented by red squares. Gene functional categories are shown by colored boxes. Isolates, loci tags, and virulence phenotype were added in the figure to facilitate comparison.

The impact of mobile insertion elements (IS) on virulence via gene interruption has been described for *F. psychrophilum*. In the strain JIP02/86, the transposition of the IS element IS256 within the collagenase gene resulted in a pseudogene formation, probably inactivating the enzymatic activity [3,50]. Beyond this, by full genome comparison, we identified 62 transposases across the five *F. psychrophilum* genomic sequences (Table S5). Eighty percent of the transpositions of these elements were in intergenic regions. For example, the high-virulent isolates FPS-S6 and 160401-1/5N and the weak-virulent strain 950106-1/1 had an element transposition between metabolic (id: E5164_11350) and T9SS sorting signal type C domain-containing (id: E5164_11355) proteins (Figure 5a). Interestingly, the moderate-virulent strain FPS-R7 and the weak-virulent strain NCIMB 1947^T^ were the only isolates with a transposition within ORFs that encoded cell-surface (id: H0H26_09915) and antibiotic ABC transporter (id: FPG3_04665) proteins, respectively (Figure 5b; Table S5).

## 4. Discussion

Bacterial pathogens exhibit significant variation in their genomic content of virulence-related factors [51]. This reflects the richness of strategies evolved by pathogens to infect host organisms [52]. In this study, we found that the nature and distribution of gene repertoires of *F. psychrophilum* isolates did not clearly reflect their virulence properties (Figure 1). The results presented here revealed a low overall genetic diversity within *F. psychrophilum* recovered from Baltic Sea countries (Figure 2 and Figure 3). However, the complete genome sequences of four selected *F. psychrophilum* isolates, 950106-1/1, FPS-R7, FPS-S6, and 160401-1/5N, obtained using Illumina and PacBio hybrid assembly, demonstrated specific dissimilarities on leucine-rich repeats (LRR) ORFs, repetitive motifs in gliding motility-associated protein, and IS transposition in these isolates (Figure 4 and Figure 5). These results suggest a link between these genetic variations and the modulation of virulence phenotypes, suggesting that these genetic regions may play an important role in the evolution and virulence of *F. psychrophilum*.

Pan-genome analyses have been an effective approach to understand pathogenic bacteria, allowing the association of genotype–phenotype profiles in specific pathogenic groups of bacteria [53,54]. Previous analysis of *F. psychrophilum* isolates from Baltic Sea countries [11] suggested that the core/pan-genome ratio (80% total gene content) was very close to those obtained from other geographical areas [8]. However, when combining genomic with virulence traits using LD_50_ in this study, there was no correlation between the presence of accessory genes and the virulence level of the isolates (Figure 1; Table S1). Similarly, the core genome composition and the virulence-related gene distribution phylogeny were not associated with virulence properties (Figure 2 and Figure 3). These results are opposite to the virulence patterns observed in *Vibrio anguillarum* [55], a pathogenic bacterium that resides in marine water and that can cause vibriosis in many fish and shellfish species [56]. The pan genome analysis of *V. anguillarum* showed a clear relationship between gene content and virulence, where the most pathogenic isolates possessed a unique accessory genome, attributed to pathogenic genomic islands, toxin-carrying prophages, and virulence-related factors [55]. Although the results obtained in this study showed that in *F. psychrophilum* the accessory gene content was not linked with virulence traits by LD_50_ (Figure 1), interesting genes were found in a genomic island of 320 kb in the moderately virulent *F. psychrophilum* isolate FPS-R7. This genomic island encoded multicopper oxidase, cysteine protease, β-lactamase, and multiple multidrug efflux pumps (Table S3). These genes have previously been connected to microbial pathogenesis, antibiotic resistance, and stress management [57,58,59,60]. However, their direct contribution in the mechanisms of pathogenicity of *F. psychrophilum* was not determined in this study; undoubtedly, these findings open new perspectives for the study of gene function in this bacterium.

The large-scale distribution and genetic homogeneity of core virulence-related factors (Table S2; Figure 3) supported the previous speculation that *F. psychrophilum* isolates all have a similar mode of pathogenicity, based on adhesion, colonization, and destruction of fish tissues [11]. An earlier report showed that all the isolates had in vitro proteolytic activities (e.g., gelatinase, caseinase, elastinase) and gliding motility except the *F. psychrophilum* isolate FPS-S11B [4], while another study established that this specific isolate had a premature stop codon in the gliding motility protein GldB, corroborating that gliding motility genes are linked to virulence-related properties in *F. psychrophilum* [12,13]. However, besides this finding, virulence-related gene profiling was not strictly linked to the virulence phenotype by LD_50_ presented by the isolates (Table S1; Figure 3). Therefore, we propose that other genetic factors could be responsible for the virulence traits in *F. psychrophilum*.

Transposase elements are among the simplest mobile genetic elements and widespread in bacteria [61]. However, it is now clear that they play an important role as bacterial mutagenic agents, enabling the host to adapt to environmental challenges [62], colonize new niches [63], and modulate virulence [64]. In this study, we found that the *F. psychrophilum* isolates 950106-1/1, FPS-R7, FPS-S6, and 160401-1/5N harbored transposase elements inserted into genic and intergenic regions (Figure 4 and Figure 5). The direct impact of these dynamic elements on the virulence of *F. psychrophilum* was not investigated in this study; however, it has been described that IS transposition into genic sequences can modulate biofilm formation and the production of extracellular polymeric substances in the pathogenic bacteria *Staphylococcus aureus* [65] and *Enterococcus faecalis* [66]. Moreover, we also found IS transposition into non-coding regions (Figure 5), a previously described mechanism for altering the expression of adjacent genes [61]. Most commonly, the effect of IS transposition is gene repression [67,68,69]; however, cases have been described illustrating gene activation [70]. Thus, we hypothesize that transposases may promote genetic and phenotypic variability in *F. psychrophilum*.

Repetitive motifs are known to occur in a wide variety of proteins present in bacteria [71]. These motifs represent an alternative module for the assembly of various multiprotein complexes, and thus, repetitive-containing proteins often participate in a wide range of functional roles, including virulence [19]. First, we found tandem leucine-rich repeat (LRR) proteins, which tended to increase in number according to the virulence phenotype of *F. psychrophilum* isolates (Figure 4a). These LRR proteins have been shown to be part of the microbial virulence-related factors involved in the interaction with host cells [72] and the invasion of mammalian host cells [73,74]. For example, the interactions of microbe–host and immune response in the human pathogenic bacteria *Streptococcus pyogenes* [75], *E. faecalis* [76], and *Listeria monocytogenes* [77] are mediated by cell surface proteins with conserved LRR motifs. Thus, we hypothesize that these LRR cell-surface proteins could be involved in the pathogenic mechanisms of *F. psychrophilum*. Second, we found a higher number of repetitive units in a gliding motility-associated protein in the high-virulent isolates 160401-1/5N and FPS-S6 (Figure 4b; Table S4). These gliding motility-related proteins have been described as facilitators of the biological function of the type IX secretion system (T9SS), which in pathogenic *Flavobacterium* species has been verified as a major virulence determinant, playing a role in motility and translocation of cell surface adhesins, peptidases, and other enzymes and virulence-related factors [7,13,78,79]. Several studies have shown that disruption or mutations of the gliding motility-related genes resulted in defects in motility [80], extracellular enzymatic activity [6], and translocation of gliding motility proteins to the cell surface [81]; thus providing a link between the virulence and motility apparatus in pathogenic members of the *Bacteroidetes* phylum. Therefore, our finding allows us to speculate that repetitive motifs can alter both the structure and function of virulence-related proteins in *F. psychrophilum*. However, there is not a direct coupling between the number of LRR and the virulence phenotypes, and more research is required to define the evolutionary trends of the repeat motif features and their potential virulence functions in this fish pathogenic species.

## 5. Conclusions

Although diverse putative virulence-related genes have been identified in genomic sequences of *F. psychrophilum*, the role played by other genetic factors in the development of BCWS and RTFS diseases is still poorly understood [3,11]. Previous work has indicated that gliding motility and proteolytic activity are required for pathogenicity in *F. psychrophilum* [4]. Although, gene repertoire and comparative genomic analyses did not reveal a clear relationship between genotype and virulence traits in the current study (Figure 1), we found that variability in the number of consecutive leucine-rich repeat (LRR) proteins, repetitive motif dynamics, and IS transposition could be key factors in understanding the evolution and virulence of *F. psychrophilum* (Figure 4 and Figure 5). This new information will form the foundation of future investigations into the pathogenicity mechanisms of *F. psychrophilum* and stimulate various experimental studies, including genetic manipulation by specific gene knock-out [82] or transposition [83], to fully understand the factors governing the virulence in this freshwater pathogen.

## Figures and Tables

**Table 1:** Genomic features of *F. psychrophilum* isolates used in this study.

Isolate	Origin	Isolation Year	Genome Size (Mb)	Genes	CDS	GC%	tRNA	Plasmid (kb)
FPS-G1	Germany	2017	2.86	2494	2423	32.4	49	3.3
FPS-F15	Finland	2017	2.86	2496	2425	32.5	49	2.1
FPS-P1	Poland	2016	2.86	2385	2344	32.5	41	No
FPS-R9	Russia	2017	2.86	2387	2347	32.6	34	No
FPS-P3	Poland	2017	2.86	2398	2358	32.4	34	No
FPS-S6	Sweden	2017	2.86	2528	2457	32.5	49	3.3
160401-1/5N	Denmark	2016	2.82	2527	2456	32.5	49	3.3
P30-2B/09	Finland	2009	2.86	2542	2471	32.5	49	3.3
F164	Sweden	1996	2.86	2489	2418	32.3	49	No
FPS-F16	Finland	2017	2.86	2496	2425	32.5	49	No
V46	Finland	2005	2.84	2294	2258	32.4	30	No
FPS-S11A	Sweden	2017	2.86	2484	2413	32.6	49	3.3
990512-1/2A	Denmark	1999	2.86	2486	2415	32.5	49	3.3
141127-1/2N	Denmark	2014	2.85	2488	2417	32.6	49	3.3
P15-8B/11	Finland	2011	2.85	2489	2418	32.5	49	2.1
160401-1/5M	Denmark	2016	2.85	2484	2413	32.5	49	3.3
FPS-F27	Finland	2017	2.86	2490	2419	32.4	49	2.1
FPS-R7	Russia	2017	3.20	2878	2806	32.6	49	No
FPS-S9	Sweden	2017	2.86	2519	2448	32.5	49	No
030522-1/1	Denmark	2003	2.86	2534	2463	32.5	49	3.3
FPS-S10	Sweden	2017	2.86	2.495	2425	32.5	49	No
010418-2/1	Denmark	2001	2.86	2546	2475	32.6	49	3.3
K9/00	Finland	2000	2.86	2496	2425	32.5	49	No
950106-1/1	Denmark	1995	2.84	2507	2436	32.6	49	3.3
NCIMB 1947^T^	USA	Unknown	2.71	2397	2305	32.6	49	No
FPS-S11B	Sweden	2017	2.86	2486	2415	32.6	49	3.3

## Data Availability

The genomic sequences of all sequenced strains have been deposited in the NCBI database under the accession numbers listed in Table S1.

## Supplementary Materials

The following are available online at https://www.mdpi.com/article/10.3390/microorganisms9081658/s1, Figure S1: *F. psychrophilum* pan, core, and accessory genome evolution according to the number of sequenced genomes, Figure S2: Schematic representation of dispensable genome in *F. psychrophilum* isolates, Figure S3: Graphic representation of mapped reads in *F. psychrophilum* isolate JIPO82/6 as a reference genome, Table S1: Characteristics of *F. psychrophilum* isolates used in this study, Table S2: Distribution of putative virulence-related factors in *F. psychrophilum* isolates, Table S3: Distribution of genomic islands in *F. psychrophilum* isolates, Table S4: Amino acid sequences of repetitive motifs in *F. psychrophilum* isolates, Table S5: Distribution of transposase genes in *F. psychrophilum* isolates.
