# Supplementary material for: Comparative Genomic Analyses of Flavobacterium psychrophilum Isolates Reveals New Putative Genetic Determinants of Virulence Traits
Source: Microorganisms. 2021 Aug 3;9(8):1658. doi: 10.3390/microorganisms9081658 (PMC8400371; doi:10.3390/microorganisms9081658)
Supplement: Supplementary file 1 [file Figure 3S.pdf › Figure 3S.pdf]

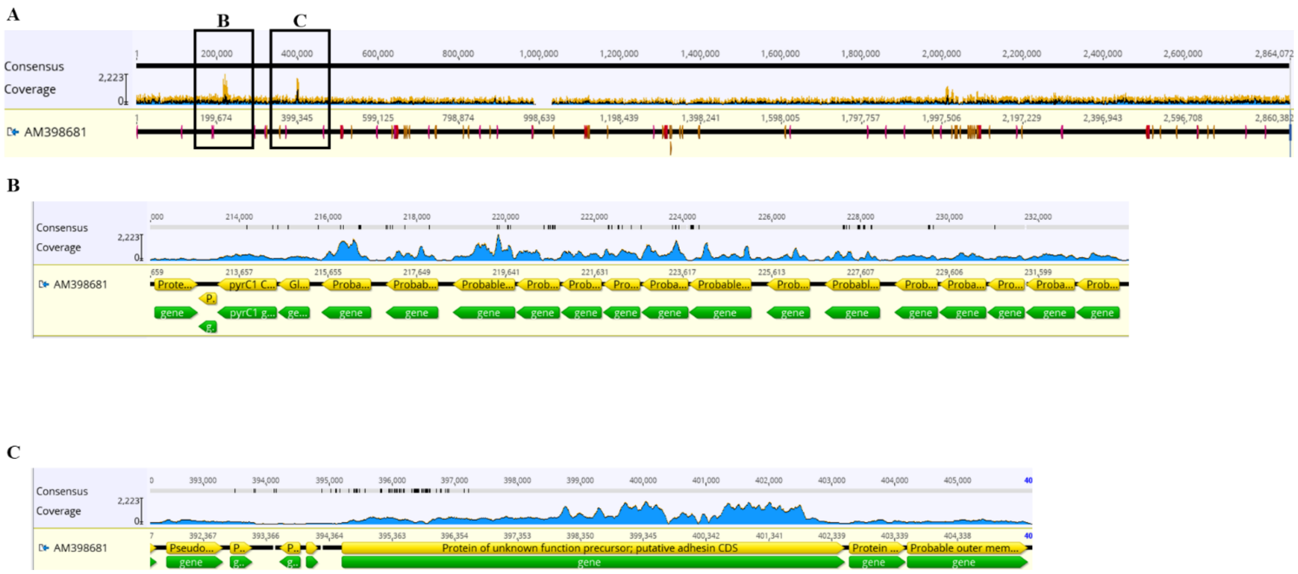

Figure 3S. **Graphic representation of mapped reads in *F. psychrophilum* isolate JIPO82/6 as a reference genome**. A) Full genome coverage by mapped reads (from Illumina sequencing technology) in the *F. psychrophilum* isolate JIPO82/6 (Accession number AM398681). Black squares highlight picks on genomic zones with high coverage (>1500x) compared with the coverage of the entire genome. B) Zoom in Leucine-rich repeat (LRR) ORFs. C) Zoom in putative adhesin or former gliding motility-associated protein. The genome coordinates, LRR and putative adhesin genes, read coverage, and mapped reads are shown in the figure. The *F. psychrophilum* isolate FPS-S6 is used as a representative model.
