# Supplementary material for: Comparative Genomic Analyses of Flavobacterium psychrophilum Isolates Reveals New Putative Genetic Determinants of Virulence Traits
Source: Microorganisms. 2021 Aug 3;9(8):1658. doi: 10.3390/microorganisms9081658 (PMC8400371; doi:10.3390/microorganisms9081658)
Supplement: Supplementary file 1 [file microorganisms-09-01658-s001.zip › Table 3S.pdf]

Table 3S. Distribution of genomic islands in *F. psychrophilum* isolates

Genomic island	Isolate	Localization	Size (kb)	Genetic feature(s)
1	FPS-R7	H0H26_09110-H0H26_09135	8.1	N-acetyltransferase (H0H26_09120) and transporter (H0H26_09110)
2		H0H26_09230-H0H26_09505	46.1	Prophage-like element
3		H0H26_10290-H0H26_10370	25.4	DNA-methyltransferase (H0H26_10320) and helicase (H0H26_10360)
4		H0H26_11345-H0H26_11810	71.2	Prophage-like element
5		H0H26_12900-H0H26_12930	10.4	Lantibiotic dehydratase (H0H26_12920) and peptidase (H0H26_12910)
6		H0H26_00790-H0H26_00875	12.2	Unknown function proteins
7		H0H26_01960-H0H26_01995	8.0	Fic protein (H0H26_01965)
8		H0H26_03655-H0H26_03720	16.9	beta-lactamase (H0H26_03705)
9		H0H26_03910-H0H26_04225	82.3	Transposases, unknown function proteins and T9SS type A sorting domain-containing protein (H0H26_04050)
10		H0H26_03730-H0H26_04870	320	Multicopper oxidase domain-containing protein (H0H26_04775), transporters (H0H26_04095- H0H26_04110), proteases (H0H26_04400), abortive infection proteins (H0H26_04445), beta-lactamase (H0H26_04585) and multiple transposases
11	NCIMB 1947^T	FPG3_04905-FPG3_04875	8.3	Deoxyribonuclease (FPG3_04875)
12		FPG3_01405-FPG3_01440	9.6	Unknown function proteins
13	FPS-S6	E5164_12690-E5164_12785	18.1	Fibrobacter succinogenes major paralogous domain-containing protein (E5164_12695) and unknown function proteins
14	160401-1/5N	E5165_12735-E5164_12785	18.1	Fibrobacter succinogenes major paralogous domain-containing

				protein (E5164_12695) and unknown function proteins
