# Supplementary material for: Comparative Genomic Analyses of Flavobacterium psychrophilum Isolates Reveals New Putative Genetic Determinants of Virulence Traits
Source: Microorganisms. 2021 Aug 3;9(8):1658. doi: 10.3390/microorganisms9081658 (PMC8400371; doi:10.3390/microorganisms9081658)
Supplement: Supplementary file 1 [file microorganisms-09-01658-s001.zip › Table 4S.pdf]

Table 4S. Amino acid sequences of repetitive motifs in *F. psychrophilum* isolates

Isolate	Locus tag	Number of repetions	Motif consensus sequence
JIPO2/86	FP2519	30	DYTTTVTTVTI.PAGQxTTVNVTVPI
		19	DTIDEPAETFTVNGTV
		6	ITTATGTAGTS
NCIMB1947^{T}	FPG3_01745	32	VNVTVxxPILEDTIDxxEPAETFxxxTVxNG
		21	TAGTSxDYTxxTTVTTxxVTxxxxxIPAGQTT
950106-1/1	H0H25_07270	51	INEGxAGTVTIpVSIDvVSSVDTVIN
		18	LEDTIDEPAETFTVxNGTVTS
FPS-R7	H0H26_09970	32	TYTLTVTNTSxNGCTSTNSVIxxVTVDKTAPTAT
160401-1/5N	E5165_12645	66	ILEDTIdePAETFTVNGTV
		21	ATIGNVTINEGAGTVTIPVSIDVVSSVDTVINITTATGTAGTSDYTTT
FPS-S6	E5164_01700	46	INEG.AGTVTIpVSIDvVSSVDTVIN
		36	DTIDEPAETFTVNGTV
		11	ITTATGTAGTS
