# Supplementary material for: Comparative Genomic Analyses of Flavobacterium psychrophilum Isolates Reveals New Putative Genetic Determinants of Virulence Traits
Source: Microorganisms. 2021 Aug 3;9(8):1658. doi: 10.3390/microorganisms9081658 (PMC8400371; doi:10.3390/microorganisms9081658)
Supplement: Supplementary file 1 [file microorganisms-09-01658-s001.zip › Table 5S.pdf]

Table 5S. Distribution of transposase genes in *F. psychrophilum* isolates

Strain	Locus tag	Transposase type	Genomic context	Functions
FPSR7	H0H26_08935	IS204 transposase	Intergenic region between H0H26_08930 and H0H26_08945 genes	Metabolic genes
	H0H26_08940	IS204 transposase	Intergenic region between H0H26_08930 and H0H26_08945 genes	Metabolic genes
950106-1/1	H0H25_00805	IS982 transposase	Intergenic region between H0H25_00800 and H0H25_00810 genes	Metabolic genes
160401-1/5N	E5165_03945	IS982 transposase	Intergenic region between E5165_03950 and E5165_03940 genes	Metabolic genes
FPS-S6	E5164_03940	IS982 transposase	Intergenic region between E5164_03945 and E5164_03935 genes	Metabolic genes
950106-1/1	H0H25_01690	ISFps1 transposase	Operon; intergenic region between H0H25_01660 and H0H25_01700 genes	Multidrug transporter
160401-1/5N	E5165_03005	ISFps1 transposase	Operon; intergenic region between E5165_03035 and E5165_02995 genes	multidrug transporter
FPS-S6	E5164_03000	ISFps1 transposase	Operon; intergenic region between E5164_03030 and E5164_02990 genes	multidrug transporter
NCIMB1947^T	FPG3_04660	Transposase	Gene FPG3_04665	Antibiotic ABC transporter ATP-binding protein
FPS-R7	H0H26_11865	IS204 transposase	Gene H0H26_11860	Hypothetical protein
	H0H26_11870	IS204 transposase	Gene H0H26_11860	Hypothetical protein
FPS-R7	H0H26_09920	IS982 transposase	Gene H0H26_09915	Transmembrane protein
950106-1/1	H0H25_02390	IS256 transposase	Intergenic region between H0H25_02385 and H0H25_02395 genes	Transporters

160401-1/5N	E5165_02290	IS256 transposase	Intergenic region between E5165_02295 and E5165_02285 genes	Transporters
FPS-S6	E5164_02285	IS256 transposase	Intergenic region between E5164_02290 and E5164_02280 genes	Transporters
NCIMB1947^T	FPG3_04005	IS4 transposase	Intergenic region between FPG3_04010 and FPG3_04000 genes	Transporters
NCIMB1947^T	FPG3_03390	IS982 transposase	Intergenic region between FPG3_03395 and FPG3_03385	Metabolic genes
FPS-R7	H0H26_13075	Transposase domain DUF772	Intergenic region between H0H26_13070 and H0H26_13080 genes	Exopolysaccharide transportation
950106-1/1	H0H25_04155	IS982 transposase	Gene H0H25_04150	Probable phage protein
FPS-S6	E5164_11140	IS982 transposase	Gene E5164_11135	Probable phage protein
950106-1/1	H0H25_04365	IS256 transposase	Intergenic region between H0H25_04360 and H0H25_04370 genes	T9SS sorting signal type C domain-containing protein
160401-1/5N	E5165_11350	IS256 transposase	Intergenic region between E5165_11345 and E5165_11355	T9SS sorting signal type C domain-containing protein
FPS-S6	E5164_11355	IS256 transposase	Intergenic region between E5164_11350 and E5164_11360	T9SS sorting signal type C domain-containing protein
FPS-R7	H0H26_00330	IS256 transposase	Intergenic region between H0H26_00325 and H0H26_00335	Metabolic genes
950106-1/1	H0H25_05350	Transposase	Operon; intergenic region between H0H25_05320 and H0H25_05360	Metabolic genes
160401-1/5N	E5165_12350	Transposase	Operon; intergenic region between E5165_12320 and E5165_12360	Metabolic genes
FPS-S6	E5164_12355	Transposase	Operon; intergenic region between	Metabolic genes

			E5164_12325 and E5164_12365	
NCIMB1947[T]	FPG3_12250	IS256 transposase	Intergenic region between FPG3_12245 and FPG3_12255 genes	Protease
FPS-R7	H0H26_01265	IS982 transposase	Intergenic region between H0H26_01260 and H0H26_01270 genes	Metabolic genes
FPS-R7	H0H26_01930	IS982 transposase	Operon; Intergenic region between H0H25_06395 and H0H26_01915	Metabolic genes
950106-1/1	H0H25_06995	IS3 transposase	Intergenic region between H0H25_06990 and H0H25_07010 genes	Hypothetical proteins
160401-1/5N	E5165_01425	IS3 transposase	Intergenic region between E5165_01420 andE5165_01440 genes	Hypothetical proteins
FPS-S6	E5164_01410	IS3 transposase	Intergenic region between E5164_01405 and E5164_01425	Hypothetical proteins
FPS-R7	H0H26_02745	IS204 transposase	Intergenic region between H0H26_02755 and H0H26_02740	Metabolic genes
950106-1/1	H0H25_07115	IS982 transposase	Operon; intergenic region between H0H25_07140 and H0H25_07105	T9SS type A sorting domain-containing protein
160401-1/5N	E5165_01540	IS982 transposase	Operon; intergenic region between E5165_01565 and E5165_01530	T9SS type A sorting domain-containing protein
FPS-S6	E5164_01535	IS982 transposase	Operon; intergenic region between E5164_01560 and E5164_01520	T9SS type A sorting domain-containing protein
950106-1/1	H0H25_07375	Transposase	Intergenic region between H0H25_07370 and H0H25_07380 genes	Metabolic genes
160401-1/5N	E5165_09870	Transposase	Intergenic region between	Metabolic genes

			E5165_09875 and E5165_09865	
FPS-S6	E5164_09875	Transposase	Intergenic region between E5164_09880 and E5164_09870	Metabolic genes
950106-1/1	H0H25_07955	IS256 transposase	Intergenic region between H0H25_07950 and H0H25_07960 genes	Metabolic genes
160401-1/5N	E5165_09295	IS256 transposase	Intergenic region between E5165_09300 and E5165_09290 genes	Metabolic genes
FPS-S6	E5164_09300	IS256 transposase	Intergenic region between E5164_09305 and E5164_09295	Metabolic genes
FPS-R7	H0H26_03555	IS256 transposase	Gene H0H26_03560	Metabolic genes
NCIMB1947^{T}	FPG3_06805	IS982 transposase	Intergenic region between FPG3_06810 and FPG3_06795 genes	virulence-associated e family protein
950106-1/1	H0H25_08575	IS982 transposase	Operon; Intergenic region between H0H25_08570 and H0H25_08595	Gliding motility genes
160401-1/5N	E5165_08755	IS982 transposase	Operon; Intergenic region between E5165_08760 and E5165_08735	Gliding motility genes
FPS-S6	E5164_08745	IS982 transposase	Operon; Intergenic region between FPG3_07330 and FPG3_07365	Gliding motility genes
NCIMB1947^{T}	FPG3_08340	Transposase	Intergenic region between FPG3_08330 and FPG3_08345	Metabolic genes
NCIMB1947^{T}	FPG3_08435	Tranposase	Gene FPG3_08440	Putative PepSY_TM-like protein
FPS-R7	H0H26_06180	IS256 transposase	Intergenic region between H0H26_06175 and H0H26_06185	Metabolic genes
NCIMB1947^{T}	FPG3_08605	IS256 transposase	Gene FPG3_08610	Hypothetical protein

FPS-R7	H0H26_06345	IS204 transposase	Intergenic region between H0H26_06340 and H0H26_06355	Ribosomal proteins
	H0H26_06350	IS204 transposase	Intergenic region between H0H26_06340 and H0H26_06355	Ribosomal proteins
NCIMB1947^{T}	FPG3_08955	IS4 transposase	Operon; Intergenic region between FPG3_08950 and FPG3_08960	Transporters
FPS-R7	H0H26_07275	IS200/IS605 transposase	Intergenic region between H0H26_07270 and H0H26_07280	Metabolic genes
950106-1/1	H0H25_10330	IS256 transposase	Gene H0H25_10325	Collagenase
FPS-R7	H0H26_07015	IS256 transposase	Gene H0H26_07010	Collagenase
160401-1/5N	E5165_07010	IS256 transposase	Gene E5165_07015	Collagenase
FPS-S6	E5164_07000	IS256 transposase	Gene E5164_07005	Collagenase
950106-1/1	H0H25_11590	Transposase	Intergenic region between repeat zone and H0H25_11595	CRISPR array
FPS-S6	E5164_12830	IS256 transposase	Intergenic region between E5164_12825 and E5164_00935 genes	Leucine-reach repeat region
