# Supplementary material for: Comparative Genomic Analyses of Flavobacterium psychrophilum Isolates Reveals New Putative Genetic Determinants of Virulence Traits
Source: Microorganisms. 2021 Aug 3;9(8):1658. doi: 10.3390/microorganisms9081658 (PMC8400371; doi:10.3390/microorganisms9081658)
Supplement: Supplementary file 1 [file microorganisms-09-01658-s001.zip › Figure 1S.pdf]

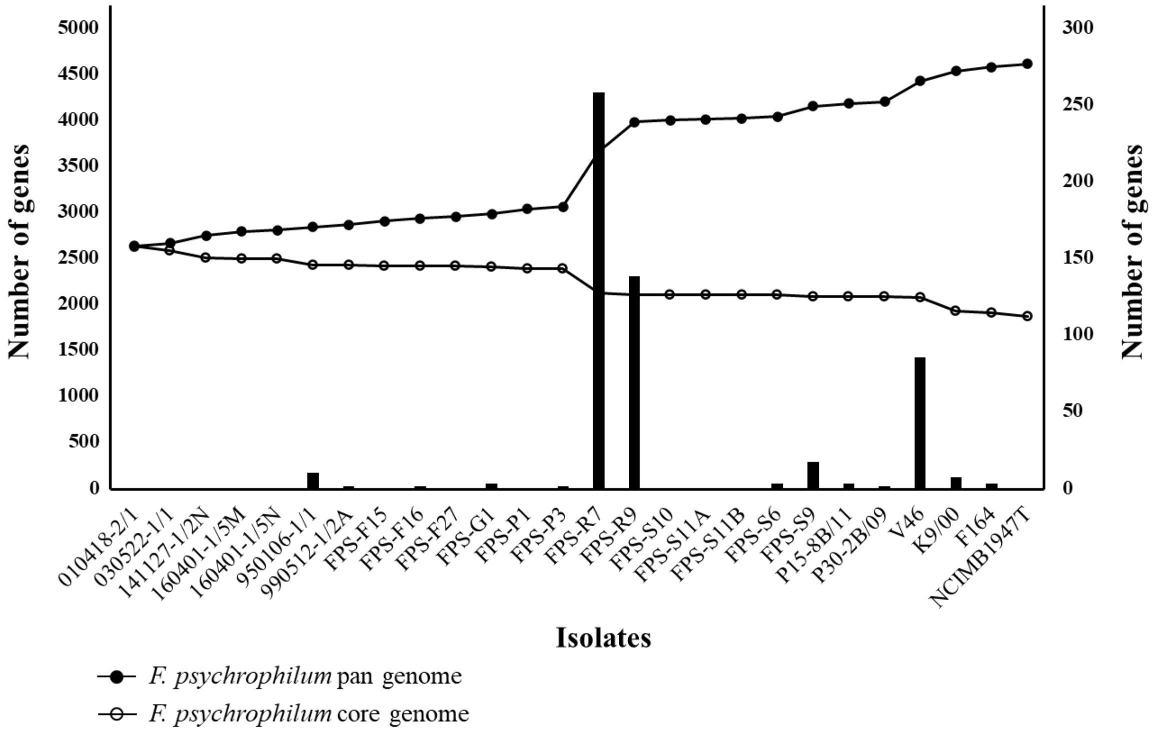

Figure 1S. ***F. psychrophilum* pan, core and accessory genome evolution according to the number of sequenced genomes**. Total number of genes (pan-genome), shared genes (core genome) and unique genes (accessory genome) for a given number of genomes sequentially added.
