# Supplementary material for: Comparative Genomic Analyses of Flavobacterium psychrophilum Isolates Reveals New Putative Genetic Determinants of Virulence Traits
Source: Microorganisms. 2021 Aug 3;9(8):1658. doi: 10.3390/microorganisms9081658 (PMC8400371; doi:10.3390/microorganisms9081658)
Supplement: Supplementary file 1 [file microorganisms-09-01658-s001.zip › Figure 2S.pdf]

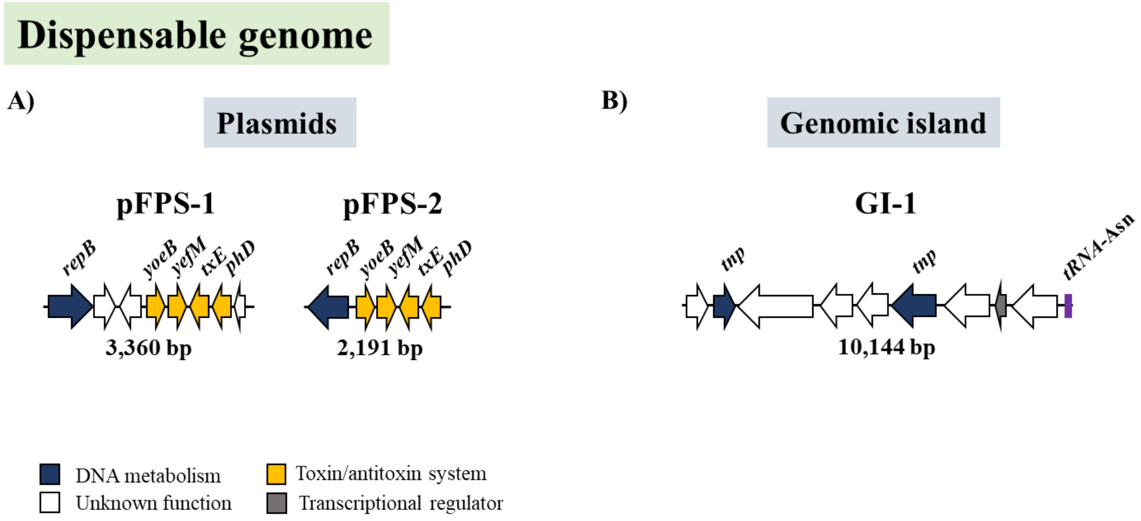


**Figure 2S. Schematic representation of dispensable genome in *F. psychrophilum* isolates.** A) Plasmids sequences of 3,3 and 2,1 kb were found in 15 out of 26 *F. psychrophilum* isolates (Table 1). B) Genomic island of 10.1 kb found in the *F. psychrophilum* isolates FPS-G1, FPS-S6, FPS-P1 and FPS-P3 (H0I59_05470-H0I59_05510). Genes are represented as arrows. Functions for genes are indicated in colored boxes
